# The Promotions of Sustainable Lunch Meals in School Feeding Programs: The Case of Italy

**DOI:** 10.3390/nu13051571

**Published:** 2021-05-07

**Authors:** Laura Rossi, Marika Ferrari, Deborah Martone, Luca Benvenuti, Alberto De Santis

**Affiliations:** 1 CREA Council for Agricultural Research and Economics—Research Centre for Food and Nutrition, Via Ardeatina 546, 00178 Rome, Italy; laura.rossi@crea.gov.it (L.R.); marika.ferrari@crea.gov.it (M.F.); deborah.martone@crea.gov.it (D.M.); 2 Department of Computer, Control and Management Engineering, Sapienza University of Rome-via Ariosto 25, 00185 Rome, Italy; alberto.desantis@uniroma1.it

**Keywords:** school meals, menus’ planning, feeding program, environmental impact, carbon footprint, mathematical modeling

## Abstract

School is considered a privileged environment for health education and school feeding represents an opportunity for promoting sustainable foods to young generations. The objective of this paper is to demonstrate that is possible to select, from existing school menus, recipes that combine healthy foods with low environmental impact. A national sample of Italian school menus was collected and a total number of 194 recipes were included on a database containing 70 first courses, 83 s courses, 39 side dishes, 1 portion of fruit, and 1 portion of bread. A mathematical model was conceived to combine nutritional adequacy and acceptability criteria while minimizing GHGs emissions. The result is a four-week menu characterized by large vegetable components that were used not only as side dishes but also as ingredients in the first and second courses. Legumes and pasta are often included, and white meat is selected instead of red meat. The findings presented in this paper demonstrated that it is possible to design environmental-friendly meals from existing school menus. The mathematical model developed in this work has the potentiality of being completely scalable, easily updatable, and widely utilizable in different settings either for design or monitoring purposes as well as for research data collection.

## 1. Introduction

Nutrition, the food system, and the environment are inextricably linked. The health of humans cannot be isolated from that of the ecosystem [1]. A “sustainable” diet promotes environmental and economic stability through low-impact, high quality, and affordable foods, improving public health through adequate nutrition [2]. Sustainable consumption and production represent an important aspect of the Sustainable Development Goals that claimed for promotion of dietary patterns able to contribute to reducing environmental, economic, and social costs, strengthening economic competitiveness, and reducing poverty [3]. Definition of a healthy diet is consensual; according to WHO [4], a healthy diet helps protect against malnutrition in all its forms, as well as against non-communicable diseases, including diabetes, heart disease, stroke, and cancer. According to Katz and Meller [5], the optimal eating pattern could be easily summarized in a catchphrase as “Food, not too much, mostly plants”. The quantitative limitation of foods and the maximization of vegetable components represent the link among nutrition recommendations and sustainability attributes. Even if the concepts seem clear, sustainability implications of the diet remain, up to now, elusive and undefined, requiring the definition of a proper set of indicators. Food production is an important contributor to greenhouse gas emissions (GHGE) that are largely used as an indicator of the environmental impact of food consumption [6]. Animal source foods produce more GHGE than plant-based foods so that several studies on diet modeling found that a healthy diet is also resulting in low GHGE [7,8].

School is a privileged environment for health and nutrition education as well as for environmental awareness as it represents one of the major contexts for learning where habits and life-styles are acquired and solidified. School meal menus are classically designed to promote healthy eating habits aimed to prevent overweight and obesity and their consequences in terms of the incidence of non-communicable diseases. School meals represent a chance to tackle all these complex issues [9]. School feeding provision in Italy has a long tradition. School meals have been shown to contribute significantly to overall dietary habits [10,11], they are a way to transmit to schoolchildren food habits in line with dietary recommendations. Italian National guidelines for school feeding prepared by the Ministry of Health [12] were conceived as an informative document with different degrees of applications at the regional level according to local autonomy. An updated revision of the school feeding guidelines in Italy was released in 2018 [13]. This revision was a cornerstone since it includes aspects of food behavior, culture, and acceptability in addition to nutritional guidelines. Moreover, aspects related to organic foods and waste prevention, and other sustainability factors are coherent with the principles of EU Green and Sustainable Public Procurement [14]. They recommend achieving a reduction of the environmental impact when public authorities procure goods and services, such as public meals. The underlying idea of this approach is related to the fact that the lunch meal should be considered part of the educational pathways in terms of Italian food culture and the environmentally friendly food system.

The public/institutional meals play an important role in contributing to sustainability because they are responsible for a significant part of many people’s food consumption, and they influence their food habits. School feeding programs are considered a target for sustainability actions. Indeed, a recent explorative review [15] claimed that schools and hospitals are the most dominant arenas where both health and sustainability have been addressed in order to reach a more holistic perspective on food consumption of public meals. In Spain, a feeding program was designed to develop a one-month healthy and sustainable lunch plan for primary school children [11] that provides a reduction of 13–24% of GHGE and 10–15% of the cost while maintaining the nutritional quality. In [10] it is shown, through an intervention study, that optimization of school meal planning can decrease GHGE at 40% and reduce costs while maintaining nutritional adequacy and acceptability for pupils. Similarly, a study performed in the UK [7] shows that healthy and lower GHGE diets could be proposed by tailoring changes to specific income groups to make dietary changes more achievable. Finally, school meal programs are of particular interest to promote changes in food choice during childhood to re-shape food habits for all the population in the long-term. Children’s behaviors and habits are more malleable than those of adults, and school meals tend to cut across socio-economic classes [16]. The promotion of dietary shift to increase sustainability aspects in public meals became of increasing importance at the level of national and local governments as demonstrated by the actions of the municipality of Barcelona that signed the “Good Food Cities Declaration” [17]. In the framework of this endorsement, it was set an intervention for the academic year 2020–2021 aimed to introduce low-carbon meals in public schools. The Authors estimated that transition to a low-carbon meal would reduce between 46% and 60% of the environmental impacts. However, the evaluation of acceptance of the new meals by scholars and the adaptation of the school kitchen staff to the new menu remain open questions.

This paper is inspired by the idea of Benvenuti et al. [18] who elaborated a mathematical model and an optimization method able to identify dietary choices with reduced environmental impact, measured as water consumed for food production and carbon footprint, whilst ensuring a proper intake of energy and nutrients. The mathematical model was tested using the recipes in the menu of the Municipality of Rome school feeding program. The study provided an early attempt to evaluate the effectiveness of the proposed approach. The model is scalable, meaning that it has the potential capacity to work with larger databases, more recipes, and ingredients as well as fixing several nutrition parameters without affecting its structure.

The present work aimed to test the effectiveness of the approach on a larger ensemble of recipes extracted from national databases, and an extended optimization model that addresses more detailed acceptability and nutritional issues.

## 2. Materials and Methods

### 2.1. School Menus, Recipes, Food Composition Data

Primary schools (children aged 6–11 years) were analyzed in consideration of the fact that the age group attending this didactical cycle is the main user of the school feeding program.

A sample of 52 Italian school menus was collected, Italian macro-regions (North, South, Centre, and Islands) were represented. From the menus, single recipes were extracted to have a list of first choices, in general including pasta or other carbohydrate sources, a list of second dishes, in general, a source of protein, a list of side dishes, fresh fruit, and bread. Tap water is the only beverage allowed for lunch. The list of recipes considered is given in the Supplementary Material. The energy and nutrients composition of the recipes were calculated using the CREA Food and Nutrition database [19] completed with the Food Composition Database for Epidemiological Studies in Italy in case of missing items [20]. Composition data were combined with GHGE using the database duly prepared using data and methodology reported by Ferrari et al. [21]. The final sample of recipes included in the model was 70 first courses, 83 s courses, 39 vegetables for a total number of 194 recipes including fruit and bread. All the recipes found in the menus were included in sample; actually, we stopped the search of addition-al menus because recipes became more and more repetitive.

Energy and nutrient intakes were established based on Italian recommendations [22], and their average values for lunch in primary school canteens were obtained considering that this meal should cover 35% of the daily amount as recommended by Italian Guidelines for healthy eating [23]. To allow nutrient intake variability, upper and lower bounds of energy and nutrient were established with ranges around the average values different for daily and weekly consumptions (Table 1).

This corresponds to a slightly larger variation on the single lunch while keeping the weekly intake closer to the average. Constraints were imposed also for fibre and sodium in addition to those fixed for macronutrients. In Italy, fibre consumption is particularly low in the considered age group [24,25], thus it is important to stress adherence to the recommendation in conceiving menus. On the other hand, restriction on sodium intake was set up considering the preventive value of early reduction of salt intake in this age group as recommended by WHO [26].

General acceptability of the menu was accomplished by making a trade-off between promoting healthy foods (i.e., fruit, vegetables, and legumes) and making menus attractive for children by avoiding the monotony of food choices. This was done also including foods that are normally less accepted by children by operating a combination of ingredients, and preparation of the dishes that improve the preference by this age group. The presence of similar foods in the same lunch and similar dishes in the same week has been limited to guarantee a varied menu. The acceptability requirement and arrangements of the different ingredients are detailed in Table 2.

### 2.2. Green House Gas Emission Data

GHGE values (kg CO_2_ equivalent/100 g product) of food items used for recipes were based on the dataset published by Ferrari et al. [21]. Matching among similar food items was carried out (e.g., orange juice GHGE value was applied for all types of fruit and vegetable juices) to cover all the ingredients of 194 recipes considered in the present paper to plan menus with sustainability characteristics.

### 2.3. Mathematical Modelling and Optimization Method

The research question is that of determining the monthly schedule for the primary school lunch with the minimum carbon footprint. The data consist of a set of recipes, whose composition and serving sizes are fixed, for which the energy, nutrient contents, and carbon footprint are available. The schedule must be organized by choosing within the given set of recipes the sequence of daily lunches to minimize the total GHG emission needed to serve them while satisfying some constraints related to proper energy and nutrients intake and food palatability. As discussed in Section 2.1, these constraints are related to the composition of the meal (first and second choices, side dish, bread, and fruit), daily and weekly established boundaries based on nutrient intake and allowed weekly and monthly repetitions of each recipe or recipes in the same food group (see Table 1 and Table 2).

The problem consists in specifying, for each one of the five days d in which the lunch is served (d=1,⋯,5) and for each one of the weeks w considered (w=1,⋯,4), the set of recipes that comprise the lunch. To this end, to each recipe i=1,…,194 a binary variable $x_{d,w}^{i}$ is assigned; it assumes value 1 if the i-th recipe is part of the lunch of the d-th day of the w-th week, and 0 otherwise.

Therefore, the quantity $Q_{d,w}^{k}\;$ of the k-th item (Energy, fat,…, CO_2_ equivalent) in the lunch of the d-th day of the w-th week is

$$Q_{d,w}^{k}=\overset{194}{\underset{i=1}{\sum }}x_{d,w}^{i}\times Q_{i}^{k}$$

where $Q_{i}^{k}$ is the k-th item content in the i-th recipe, as reported in the Supplementary Material.

Therefore, the quantity $Q_{w}^{k}$ of the k-th item in the w-th week and the quantity $Q^{k}$ in the whole month are:

$$Q_{w}^{k}=\overset{5}{\underset{d=1}{\sum }}{\;Q}_{d,w}^{k},{\;Q}^{k}=\overset{4}{\underset{w=1}{\sum }}Q_{w}^{k}$$

As previously discussed, quantities $Q_{d,w}^{k}$ and $Q_{w}^{k}$ are bounded by lower and upper limits, see Table 1, and this restricts the possible choice of recipes for each lunch.

Moreover, besides nutritional requirements, the schedule must be varied and acceptable. Variety can be achieved by limiting the repetition of each dish in the week and the month. The number of times that each recipe i is served within the w-th week and in the whole month are:

$$R_{w}^{i}=\overset{5}{\underset{d=1}{\sum }}x_{d,w}^{i}\;,{\;R}^{i}=\overset{4}{\underset{w=1}{\sum }}R_{w}^{i}$$

so that variety can be ensured by fixing lower and upper values for $R_{w}^{i}$ and $R^{i}$.

On the other hand, acceptability can be achieved by limiting the repetition in the week and the month of recipes in the same food category. The number of times that recipes in category C (Rice, Vegetables,…) are served within the w-th week and in the whole month is:

$$R_{w}^{C}=\underset{i\in C}{\sum }R_{w\;}^{i},{\;R}^{C}=\underset{i\in C}{\sum }R^{i}$$

so that acceptability can be ensured by fixing lower and upper values for $R_{w}^{C}$ and $R^{C}$ (see Table 2).

The schedule is then constructed to minimize the objective $Q^{{\mathrm{CO}}_{2\mathrm{eq}}}$, that is the total amount of CO_2_eq emission. The objective is a linear function of the 3880 binary variables $x_{d,w}^{i}$ as follows

$$Q^{{\mathrm{CO}}_{2\mathrm{eq}}}=\overset{4}{\underset{d=1}{\sum }}\overset{5}{\underset{w=1}{\sum }}x_{d,w}^{i}\times Q_{i}^{{\mathrm{CO}}_{2\mathrm{eq}}}$$

where $Q_{i}^{{\mathrm{CO}}_{2\mathrm{eq}}}$ is the GHGE amount needed to serve the i-th recipe. As said above, lower and upper bounds for $Q_{d,w}^{k}$, $Q_{w}^{k}$, $R_{w}^{i}$, $R^{i}$, $R_{w}^{C}$ and $R^{C}$, define constraints on the variables $x_{d,w}^{i}$.

Note that the problem is not a classical Linear Programming optimization since the variables may assume only two values (0 or 1) and do not vary continuously in a range. Therefore, the problem is a constrained Linear Integer Programming one. The optimization was carried out by using the online version of CPLEX for AMPL on the NEOS server (https://neos-server.org/neos/ (accessed on 3 December 2020)); the model is extensively described in the paper of Benvenuti et al. [27].

## 3. Results

The result of the optimization procedure is a 4-week menu with the lowest GHGE and complying with the nutritional constraints and acceptability restrictions previously described. Table 3 shows the dishes’ composition and their sequence in the menu.

**Figure 1:**
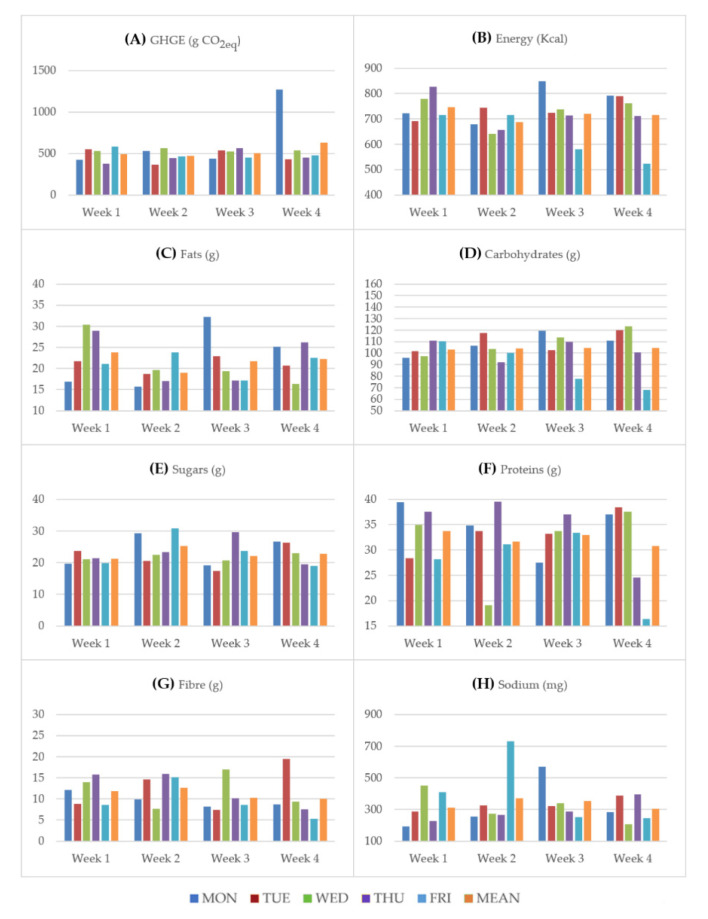
GHGE, energy and nutrient contents for each lunch and weekly average.

The average optimized meal is characterized by the following energy and nutrient contents: 718 kcal of energy, 104 g of carbohydrates, 32.3 g of protein, 21.7 g of fat, 22.8 g of sugar, 11.2 g of fibre, and 335 mg of sodium with a resulting average CO_2_ equivalent of 525 g. The meal plan has a large component of vegetables both as side dishes and as ingredients of other courses. The first courses of the menu often contain legumes while the second courses often consist of white meat (e.g., poultry and turkey). Figure 1 reports GHGE, the energy and nutrient contents of each lunch and the weekly average. It is then easy to check that the lower and upper boundaries of Table 1, corresponding to dietary recommendations, are satisfied. As shown in Figure 1A, the distribution of the GHGE (g CO_2_ equivalent per meal) is quite homogenous but there is a clear peak on the fourth week in which a recipe with red meat (meatballs with tomato) is proposed. The distribution of energy and nutrients across the weeks is reported in the other panels of the figure. Energy (Figure 1B) has a peak on the third week (Monday) due to the combination in the same lunch of filled pasta with butter, an energy-rich dish, and potatoes, resulting in a double presence of carbohydrate source foods in the same meal (see also Figure 1D). Of note, this combination results also in a peak of fat content as shown in Figure 1C. The other two peaks of energy (Figure 1B) and fats (Figure 1C) on the first week (Wednesday and Thursday) depend on recipes with cheese and lamb, both sources of fat and consequently of energy. Fridays of the third and fourth weeks are characterized by the lowest content of energy and carbohydrates (see also Figure 1D). The lunches on Mondays of the first and second weeks are particularly low in fat contents due to the presence of white meat and legumes (Figure 1C). The distribution of carbohydrates (Figure 1D) is quite homogenous apart for Fridays of the third and fourth weeks in which the presence of soups lowers the net quantity of rice and pasta that are combined with other ingredients. Sugar contents are reported in Figure 1E.

Carrots, which contain a high quantity of sugar compared to other vegetables, are responsible for the peaks observed on the second (Monday and Friday) and third (Thursday) weeks. Figure 1F shows the distribution of proteins. The second week (Wednesday) and the fourth week (Friday) have the lowest quantities of protein because on these days only 1 egg is considered as the second course. This corresponds to a small portion that compensates for the relatively high content of proteins in the eggs. Cheese is another possible second-course providing fats and lowering the quantity of protein (Friday of the fourth week). The two relevant peaks of proteins on Monday of the first week and Thursday of the second week are due to the presence of legumes with pasta as the first course, and meat as the second course. In general, the presence of legumes is responsible for a high quantity of proteins also on the other days (e.g., Thursday on the first week and Tuesday on the fourth week) even though legumes represent proteins from a vegetable source. In addition to that, legumes are also an important contributor to the fibre content of the proposed menu (Figure 1G), as shown by the two peaks in the third and fourth week (Wednesday and Tuesday, respectively).

The sodium contents are reported in Figure 1H. The two peaks in the second and third week (Friday and Monday, respectively) could be explained with the presence of pasta filled with processed (and salty) meat in the first case and with the presence of semi-hard cheese in the second case, both considerable sources of salt.

**Figure 2:**
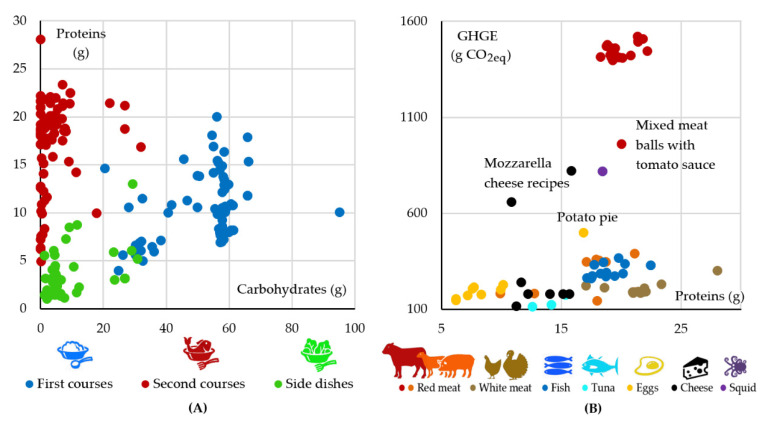
(**A**) Carbohydrate and protein contents of the recipes; (**B**) protein contents and GHGE of the second-course recipes.

Figure 2 shows some characteristics of the sample of 194 recipes used to design the menu. In more detail, Figure 2A displays the carbohydrate and protein contents of the recipes analyzed. As expected, the second courses are the main contributors of proteins while the first courses are the main contributors of carbohydrates. However, it should be pointed out that proteins provided by first courses are a significant source of vegetal proteins coming from legumes consumed with pasta or rice. On the other hand, Figure 2B shows the relation between proteins contained in the second-course recipes and their GHGE.

Unsurprisingly, the optimization procedure does not include in the optimized menu recipes having beef as an ingredient considering its high emissions. Dishes with pork and lamb were preferred instead, because of their lower values of GHGE. However, since, according to acceptability requirements, beef must be included in the menu at least once a month, the procedure selected the only recipe that contains beef together with pork (mixed meatball) resulting in attenuated values of GHGE with respect to recipes with only beef. Considering recipes with fish, the procedure often selected those with tuna that have a lower level of GHGE. Similarly, the procedure excluded recipes with mozzarella cheese since they have the highest GHGE among dairy products. Finally, recipes with eggs and vegetables were selected instead of those with eggs and processed meat or mozzarella cheese (potato pie).

## 4. Discussion

Sustainability is a concept related to the whole food chain. However, it is important to improve the sustainability level in particular within school feeding programs. Indeed, school meals can be considered as a tool for promoting healthy and sustainable food behaviors within the population in the long-term. The Italian guidelines for school meal programs [13] include in fact sustainability elements in the recommendations. The results presented in this paper support the idea that an appropriate design of school menus represents a suitable approach for the development of feeding plans aimed to improve the healthiness of meals as well as to select sustainable recipes with reduced GHGE.

The menu resulting from the process of optimization consists of foods with high nutritional quality in balanced quantities and with minimal GHG emissions.

The proposed menu design could be an important tool in the framework of policy options for increasing the sustainability of the national school feeding program. The added value of this method is related to the fact that the GHGE dataset was developed on the basis of a literature review on local data and was used to optimize the carbon footprint for the national diet using the Italian food consumption data [21]. The results of this study can be of great interest for different stakeholders in school-feeding activities as well as to some other food service areas such as company service canteens, chain restaurants, or other individual establishments. Moreover, healthy and sustainable food plans are of particular relevance nowadays within the European Green Deal, a plan to make the EU’s economy sustainable also with reduced greenhouse gas emissions [28].

The presented approach to improve school feeding programs sustainability is a concurrent factor together with several activities such as the establishment of school gardens educational activities aimed to increase students ecological understanding; programs aimed to increase fruit and vegetable consumption; actions dedicated to reduction and prevention of food waste; projects to support local farmers [29]. However, such programs can be expensive, need a sufficient logistics network and their cost-effectiveness should be evaluated [16]. On the other hand, the key feature of the present proposal is related to the fact that the use of the optimization tool represents a way to reduce the GHG emission of school food choices at limited costs.

The carbon footprint of the proposed menu is very similar to that obtained in [10] for a four-week menu for primary schools in Sweden, resulting 497 g of CO_2_eq. That menu was obtained in two steps: first, it was determined an optimized list of food quantities by linear programming, and then this list was developed into a realistic menu by a professional meal planner. Hence, the help of an experienced and creative meal planner is necessary. Moreover, acceptability is taken into account by minimizing the relative deviation of the food amounts from baseline reference values. Even if the approach is totally different from that here proposed, the amount of energy and nutrient contents, as well as that of GHGE, are very close. Anyway, the advantage of our design over that in [10] is twofold since it produces directly a realistic menu and allows managing acceptability by defining appropriate repetitions of dishes in the menu.

A further comparison can be made for the four-week menus proposed in [11] for primary schools in Spain in which an approach very similar to that here proposed is used. Nevertheless, the menus are obtained by minimizing the relative deviations of carbon footprint and cost from several pre-set target values. As a result, the amounts of CO_2_eq of those menus are higher than our, more than doubled.

The optimized menu presented in this paper is nutritionally adequate due to lower and upper bounds imposed on energy and nutrient contents. For instance, recipes with a very high content of salt (e.g., Quiche Lorraine and pizza) have not been chosen due to the upper bound on sodium. The variety of the menu can be improved by adding appropriate further bounds on the repetitions of those dishes that appear too often. For example, the repetitions of tuna, which is served for three weeks, can be reduced to one or two. The main limitation of this study is that considers GHGE as the only indicator of sustainability thus providing a partial view of the problem. However, this is quite common in the current literature and a further evolution of the program could get into consideration other aspects of the menu rather than only the minimization of GHGE.

The proposed menu has a large quota of vegetable products, white meats, and legumes. This is not only important in terms of GHGE reduction but it is a key feature also of healthy diets as widely recommended. Indeed, the Italian school menus are conceived on the basis of Mediterranean diet principles. The optimization reinforced some characteristics of the Mediterranean diet that according to the classical definition [30] is characterized by abundant plant foods (fruit, vegetables, bread, other forms of cereals, potatoes, beans, nuts, and seeds), fresh fruit as typical daily dessert, olive oil, dairy products, and fish and poultry consumed in low to moderate amounts, zero to four eggs consumed weekly, red meat consumed in low amounts. A study of guidelines for sustainability [31] showed that the three most recommendations in that sense were more plant foods, less meat, and reduction of food waste. The menu presented in this work is in line with all these aspects and it represents an important element of congruence among healthy nutritional choices and sustainability, as claimed by the EAT-Lancet Commission on healthy diets and sustainable food systems [32].

It is difficult to provide comparisons of our menus with the EAT-Lancet dietary pattern that is intended for adults having a 2500 kcal daily energy need. In addition to that, EAT-Lancet recommendations have not a prescriptive intent but represent a general model that the Authors reported as a universal healthy diet. Comparison between the recommended portions of the Italian dietary guidelines for healthy eating and the planetary healthy diet adapted to Italian habits was carried out in [33]. The Italian recommendations suggest a higher amount of fruit and vegetables compared to the planetary healthy diet, while the EAT-Lancet plan was higher in nuts and legumes, which represent the main protein sources. It seems reasonable that our menu, developed on the basis of Italian dietary guidelines, even if designed for school-age children and covering only lunch, maintains the same differences.

Nutritional constraints and GHGE minimization resulted in a menu with a big amount of vegetable proteins. This point is of public health relevance considering the importance given to the vegetable source of proteins in the very recent systematic reviews and meta-analysis of prospective cohort studies [34,35,36]. These studies demonstrated that a high intake of plant protein was associated with a lower risk of all-cause and cardiovascular disease mortality and that the replacement of foods high in animal protein with plant protein sources could be associated with longevity. The menu presented in this paper is a way to encourage a vulnerable group of the population having a low intake of legumes (e.g., children) to increase their plant protein intake to potentially decrease disease risk factors exposure in later life educating them as future adults.

However, the concept of “sustainable diet” is broad and complex, encompassing the entire food supply chain, and takes account of health, environment, affordability, and culture [37]. A key point of the present study is that recipes usually served in Italian schools were included in the optimization process. However, to the best of our knowledge, the level of acceptability of school menus in Italy is quite low with a large production of food waste. The issue of food waste in the school canteen in Italy needs to be better quantified and better addressed with preventive strategies having measurable impacts. According to Martone et al. [38], the average percentage of food wasted in Italian school canteens is 35.8%. Wasted food comes mainly from side dishes (41.5%), and then second courses (37%) and first courses (29%). Another study considering a sample of primary school canteens within the Bologna province showed that wasted and non-served foods account for 22.0% and 19.2% of prepared food, respectively [39]. As shown in [40,41,42], the waste of foods in some food groups makes the meals inadequate in terms of energy and nutrients contents and increased environmental impact. An interesting lesson learned could be the experience of OPUS project (Optimal well-being, development, and health for Danish children through a healthy New Nordic Diet) showing that children’s likings can be used as a strategy to reduce waste even if some waste is inevitable in a school setting [43]. It is then quite clear that understanding the factors determining the refusal of food by school children becomes of fundamental importance in the context of the present study. These factors could allow defining a more appropriate set of recipes and acceptability requirements to reduce food waste while complying with Italian guidelines for school lunch.

## 5. Conclusions

This study shows how a school lunch monthly plan with minimal environmental impact in terms of GHGE could be set up with the current collection of recipes. This result could at least be exploited to provide a set of general recommendations for people directly involved in school feeding programs management. The result is particularly relevant considering the potential future role of school meals as a viable tool for promoting healthy and sustainable food behaviors. Since the food habits of children are more malleable than those of adults, the selection of school meals with reduced environmental impact could have an impact on the sustainability of the overall food system in the long-term. The result could moreover be improved by considering ad-hoc recipes with suitable nutritional contents and with a low environmental impact. Further, the proposed procedure can be easily applied to some other food service areas such as company service canteens, chain restaurants or other individual establishments. The special feature of the tool, as conceived, is that the model is completely scalable and can be easily updated as well as widely utilized in different settings either for design or monitoring purposes as well as for research data collection.

## Figures and Tables

**Table 1:** Energy and nutrient established constraints by lunch for school-age children (6–11 years).

	Daily Values		Weekly Values	
	Lower	Upper	Lower	Upper
Energy (kcal)	400	900	500	800
Carbohydrates (g)	50	160	66	106
Protein (g)	15	40	21	34
Fat (g)	10	40	17	27
Sugar (g)	0	40	15	30
Fibre (g)	0	30	4	15
Sodium (mg)	100	800	300	400

**Table 2:** Acceptability requirements and ingredients distributions in the four weeks menu.

Each Lunch Must Have a Fixed Composition:	First Course	Second Courses	Vegetables
A first course, including pasta or other carbohydrate source food	Tomato pasta, soup with pasta, rice, and soup with rice must be present at least once but no more than twice a week	Red meat must be present at most once a week and at least twice but no more than three times a month	Cooked vegetables and legumes must be present at least once but no more than twice a week
A second course, in general, a source of protein	Stuffed pasta and backed pasta must be present at least once a month	Beef must be present at least once a month	Raw vegetables or salad must be present at least twice but no more than three times a week
A portion of vegetables		White meat and fish must be present at least once but no more than twice a week	Potatoes must be present no more than once a week
A portion of fruit		Cod must be present at least once a month	
A portion of bread		Eggs must be present at least once but no more than twice a week and no more than six times a month	
		Cheese must be present at most once a week and at least once but no more than three times a month	

Recipes belonging to first courses, second courses, and cooked vegetables cannot be served more than once across the month. All the other side dishes other than cooked vegetables can be served at most twice a month and no more than once a week. Processed meat recipes are not allowed on the menu.

**Table 3:** The optimized menu.

Monday	Tuesday	Wednesday	Thursday	Friday
First Week				
Mashed lentils with pasta	Pasta with tomato sauce	Rice and spinach soup	Pasta with mashed beans	Risotto with artichokes
Roasted turkey breast	Omelette with spinach	Caciotta cheese	Roasted lamb with potatoes	Tuna pie with potatoes and eggs
Crispy baked potatoes	Mushrooms with parsley	Chickpeas with tomato sauce	Fennel salad	Green salad
Bread	Bread	Bread	Bread	Bread
Fruit	Fruit	Fruit	Fruit	Fruit
**Second Week**				
Pasta with tomato sauce garlic and oregano	Vegetable soup with beans and Parmigiano	Risotto with endive	Pasta with mashed chickpeas	Vegetable soup with rice
Roasted chicken breast	Tuna with olive oil	Omelette	Turkey breast with butter and sage	Provolone cheese
Carrots with olive oil and lemon	Crispy baked potatoes	Mixed salad (winter)	Roasted peas	Carrots with butter
Bread	Bread	Bread	Bread	Bread
Fruit	Fruit	Fruit	Fruit	Fruit
**Third Week**				
Tortellini with butter and sage	Risotto with safron	Barley with mashed potatoes	Pasta with tomato sauce and basil	Pasta with potatoes
Scrambled eggs	Roasted lamb with rosemary	Baked breaded cod sticks	Strips of chicken with flour and broth sauce	Turkey breast with butter and sage
Roasted new potatoes	Spinach with olive oil	Roasted peas	Carrots with olive oil and lemon	Mixed salad (winter)
Bread	Bread	Bread	Bread	Bread
Fruit	Fruit	Fruit	Fruit	Fruit
**Fourth Week**				
Baked pasta with béchamel sauce	Pasta with peas	Pasta with tomato sauce garlic and parsley	Risotto with mushrooms	Rice and Parmigiano soup
Mixed meat balls with tomato sauce	Tuna salad	Roasted turkey	Crescenza cheese	Omelette with field herbs
Chard with olive oil	Chickpeas with tomato sauce	Roasted new potatoes	Fennel salad	Green salad
Bread	Bread	Bread	Bread	Bread
Fruit	Fruit	Fruit	Fruit	Fruit

## Data Availability

The data are available in the Supplementary Material.

## Supplementary Materials

The following are available online at https://www.mdpi.com/article/10.3390/nu13051571/s1.
