# Supplementary material for: The Promotions of Sustainable Lunch Meals in School Feeding Programs: The Case of Italy
Source: Nutrients. 2021 May 7;13(5):1571. doi: 10.3390/nu13051571 (PMC8151658; doi:10.3390/nu13051571)
Supplement: Supplementary file 1 [file nutrients-13-01571-s001.zip › nutrients-1164894-supplementary.pdf]

Sequential number	Dished for primary school		NUTRITIONAL CONTENT AND GHGs VALUE							
			**ENERGY (Kcal)**	**LIPIDS (g)**	**CARBOHYDRATES (g)**	**TOTAL SUGAR (g)**	**PROTEINS (g)**	**FIBRE (g)**	**SODIUM (mg)**	**CO2eq (g)**
1	Meat ravioli with tomato sauce	1	355.61	12.24	50.60	5.33	13.83	2.17	283.30	287.03
2	Pasta with mashed chickpeas	2	250.71	6.48	40.60	3.42	10.02	5.38	12.65	89.13
3	Pasta with mashed beans	3	246.81	5.19	41.77	3.36	10.83	6.55	12.05	125.13
4	Pasta with mashed lentils	4	243.21	4.89	28.06	2.85	10.56	5.44	13.25	89.13
5	Pasta with mashed vegetables (summer)	5	201.66	6.24	31.40	5.39	6.89	3.00	75.25	135.06
6	Pasta with mashed vegetables (winter)	6	204.56	6.25	32.02	5.71	7.02	3.15	68.75	133.76
7	Baked lasagna	7	448.41	17.47	56.17	6.91	20.00	3.02	107.70	765.98
8	Pasta with butter and parmigiano	8	353.86	11.57	55.48	3.05	10.39	1.89	51.50	182.76
9	Pasta with pesto	9	338.60	9.23	57.87	3.92	9.26	2.54	3.43	158.89
10	Pasta with tomato sauce	10	322.66	6.80	58.48	6.05	10.42	2.85	48.05	250.48
11	Pasta with tomato sauce and basil	11	320.51	6.79	58.06	5.63	10.32	2.66	40.50	249.28
12	Pasta with ragout	12	342.61	7.66	58.48	6.05	13.46	2.85	56.00	482.23
13	Pasta with vegetable ragout	13	326.36	6.82	59.27	6.83	10.55	3.17	58.95	252.15
14	Pasta with tomato souce and organic trout	14	318.26	5.84	57.77	5.34	12.15	2.61	18.40	264.68
15	Pasta with tuna	15	347.86	7.90	57.77	5.34	14.89	2.61	89.00	319.28
16	Pasta with vegetables	16	324.86	6.82	58.76	6.31	10.68	3.09	52.45	258.48
17	Pasta with tomato souce and bacon	17	371.73	10.94	58.06	5.63	13.75	2.66	323.40	359.06
18	Pasta with tomato sauce garlic and parsley	18	299.86	5.38	57.77	5.34	8.59	2.61	10.00	219.28
19	Pasta with tomato sauce garlic and oregano	19	299.86	5.38	57.77	5.34	8.59	2.61	10.00	219.28
20	Pasta with eggs and tomato sauce	20	295.16	7.26	49.85	4.31	10.59	2.88	55.45	214.28
21	Pasta with zucchini	21	308.11	6.43	56.22	3.75	9.88	2.42	42.10	154.88
22	Pasta with potatoes	22	200.76	6.29	31.90	2.43	6.06	1.78	42.95	127.48
23	Pasta with tomato sauce and anchovies	23	315.31	6.22	57.79	5.36	10.53	2.61	46.00	236.31
24	Pasta with tomato sauce and ricotta	24	332.51	7.58	59.18	6.75	10.50	2.85	33.65	285.33

25	Risotto with butter and Parmigiano	25	342.66	10.80	57.02	0.32	7.94	0.35	55.00	273.76
26	Risotto with potatoes	26	195.96	5.96	32.56	1.26	5.01	1.12	44.45	166.48
27	Risotto with tomato sauce	27	311.46	6.03	60.02	3.32	7.97	1.31	51.55	341.48
28	Risotto with safron	28	292.51	5.62	57.20	0.50	6.91	0.40	36.80	229.88
29	Risotto with mashed peas	29	306.91	5.68	59.76	1.32	7.99	1.96	62.60	285.48
30	Risotto with endive	30	297.31	5.71	58.01	1.31	7.18	0.88	39.80	235.88
31	Risotto with pumpkin	31	297.91	5.65	58.25	1.25	7.24	0.79	37.10	241.88
32	Risotto with zucchini	32	295.81	5.65	57.62	0.89	7.30	0.76	43.40	241.88
33	Tortellini with butter and Parmigiano	33	456.86	20.31	55.01	2.54	16.89	1.80	413.20	212.41
34	Pasta with butter, Parmigiano and tomato sauce	34	352.61	8.82	61.25	6.59	10.78	2.70	39.95	284.39
35	Risotto with mushrooms	35	285.83	4.47	57.27	0.57	7.70	0.63	26.58	264.25
36	Pasta with peas	36	223.45	5.71	38.33	4.02	7.11	5.03	66.60	206.40
37	Potato gnocchi with ragout	37	410.52	10.04	65.89	4.62	17.90	5.05	80.45	830.16
38	Risotto with lemon	38	294.57	5.62	57.67	0.97	6.98	0.41	37.50	237.21
39	Tortellini with tomato sauce	39	420.15	14.99	58.46	5.98	16.36	2.91	403.70	269.68
40	Vegetable soup with rice	40	264.38	4.91	46.68	2.06	11.26	6.25	36.60	186.99
41	Pasta with cured ham	41	361.76	10.26	58.52	3.86	12.46	1.98	125.25	224.68
42	Pasta with cheeses	42	364.16	10.11	56.53	4.10	15.26	1.89	156.35	309.08
43	Rice and Parmigiano soup	43	179.30	7.83	24.78	0.48	3.99	0.21	33.58	131.38
44	Potato gnocchi with tomato sauce	44	370.62	8.33	65.89	4.62	11.81	5.05	64.55	366.66
45	Risotto with vegetables	45	325.00	6.96	61.32	2.85	8.16	2.68	84.56	296.22
46	Pasta with tomato and mozzarella	46	352.52	9.05	58.50	6.06	12.91	2.98	53.15	313.26
47	Tortellini with butter and sage	47	431.54	18.72	54.97	2.50	14.18	1.80	364.92	158.69
48	Vegetable soup with beans and Parmigiano	48	303.22	6.71	49.86	3.58	13.86	7.96	35.75	169.56
49	Pasta with aubergine	49	318.67	6.38	58.81	6.38	10.04	3.65	38.40	288.89
50	Vegetable soup (summer)	50	183.88	4.93	30.17	2.12	6.61	2.72	32.90	107.27
51	Rice salad with vegetables, tuna and eggs	51	380.94	11.68	59.77	1.77	12.93	1.83	138.70	393.82

52	Risotto with artichokes	52	297.70	5.42		58.25	1.31	7.66	2.61	84.10	335.34
53	Pasta salad with vegetables, tuna and eggs	53	384.74	12.39		56.32	3.89	15.46	2.19	73.55	352.92
54	Pasta with vegetables, butter and Parmigiano	54	313.98	4.80		60.53	6.79	10.85	4.37	53.81	290.79
55	Rice and peas soup	55	181.92	5.13		30.25	2.47	5.62	3.46	74.15	239.64
56	Pasta with pumpkin	56	323.50	7.45		57.76	4.73	10.02	2.72	33.90	164.55
57	Potato gnocchi with tomato sauce	57	370.62	8.33		65.89	4.62	11.81	5.05	64.55	366.66
58	Polenta with ragout	58	404.30	10.52		66.14	2.70	15.33	2.93	51.20	606.20
59	Potato gateau	59	279.35	12.35		32.40	0.90	11.46	2.88	183.30	320.35
60	Baked pasta with béchamel sauce	60	340.30	7.69		60.65	4.50	10.91	2.04	47.35	199.33
61	Pasta capers and olives	61	324.61	8.69		56.71	4.28	8.35	2.69	57.31	177.25
62	Barley with mashed potatoes	62	219.01	6.62		35.47	0.32	6.48	4.04	36.50	142.93
63	Rice and spinach soup	63	170.21	5.49		26.19	0.36	5.58	1.23	66.90	285.63
64	Rice and cabbage soup	64	215.01	6.13		36.13	1.33	5.96	2.27	46.10	177.63
65	Risotto with mashed leeks	65	311.39	5.69		60.67	3.96	8.17	2.19	54.80	257.37
66	Pizza with mozzarella and tomato sauce	66	487.80	10.08		95.22	23.22	10.08	6.84	1774.80	234.00
67	Quiche lorraine with vegetable	67	431.53	32.86		20.50	0.97	14.64	1.72	505.55	523.19
68	Semolina dumpling	68	392.49	17.70		45.61	8.11	15.61	1.80	146.60	420.09
69	Rice with tuna and anchovies	69	348.04	8.45		57.07	0.37	14.49	0.35	353.38	337.44
70	Baked pasta with ham and cheese	70	454.90	19.77		54.54	5.88	18.06	2.22	283.95	449.58
71	Roasted lamb with potatoes	71	351.51	18.41		26.85	0.60	21.15	2.40	89.70	293.43
72	Roasted lamb with rosemary	72	179.06	11.92		0.00	0.00	18.00	0.00	79.20	165.08
73	Roasted beef	73	133.86	5.65		1.09	1.09	19.83	0.39	49.70	1399.74
74	Roasted pork	74	190.76	12.91		0.00	0.00	18.72	0.00	53.10	335.18
75	Roasted turkey	75	137.46	5.11		1.09	1.09	21.81	0.39	59.60	178.44
76	Boiled beef with balsamic vinegar	76	155.74	9.13		0.01	0.01	18.28	0.00	48.10	1399.72
77	Roasted chicken leg	77	233.96	13.45		0.00	0.00	28.05	0.00	100.50	288.68
78	Breaded pork cutlet	78	237.71	14.14		7.00	0.45	21.12	0.34	105.54	376.58
79	Croquettes of cod fillets	79	151.17	5.21		7.71	0.36	18.79	0.34	103.06	525.90

80	Croquettes of hake fillets	80	151.17	5.21	7.71	0.36	18.79	0.34	103.06	380.70
81	Turkey breast with lemon	81	145.90	5.12	3.05	0.07	22.06	0.12	46.88	181.72
82	Roasted turkey breast	82	132.26	5.08	0.00	0.00	21.60	0.00	46.80	175.88
83	Gratin dab fillets	83	140.86	5.52	3.61	0.34	19.48	0.17	150.00	360.98
84	Breaded dab fillets	84	190.22	7.24	9.45	0.71	22.47	0.46	204.66	417.08
85	Gratin cod fillets	85	126.56	4.75	3.50	0.23	17.61	0.17	92.80	506.18
86	Gratin hake fillets	86	126.56	4.75	3.50	0.23	17.61	0.17	92.80	360.98
87	Breadel sea bass fillets	87	190.22	7.24	9.45	0.71	22.47	0.46	204.66	386.28
88	Veal fillet with lemon	88	152.72	6.49	4.57	0.11	19.32	0.17	80.22	1387.94
89	Chicken breast with basil and parsley sauce	89	171.52	7.33	4.81	0.35	21.96	0.58	31.19	211.52
90	Spinach omelette	90	120.77	9.19	1.20	0.18	8.33	0.72	109.30	270.49
91	Hake fillets with tomato sauce	91	121.26	4.91	1.50	1.50	17.76	0.45	79.30	425.48
92	Cod fillets with tomato sauce	92	121.26	4.91	1.50	1.50	17.76	0.45	79.30	570.68
93	Turkey breast with butter and sage	93	134.20	5.25	0.06	0.06	21.64	0.00	47.15	186.00
94	Veal cutlet	94	154.21	5.62	5.45	0.35	20.83	0.27	124.54	1415.24
95	Omelette with field herbs	95	111.57	9.19	0.00	0.00	7.21	0.00	86.50	159.29
96	Cuttlefish with peas	96	157.46	5.95	8.07	3.72	18.46	4.17	173.90	924.08
97	Zucchini omelette	97	115.97	9.23	0.56	0.52	7.73	0.48	95.30	175.29
98	Ham omelette	98	143.82	11.39	0.14	0.14	10.18	0.00	183.70	224.54
99	Scallops with tomato sauce	99	180.32	9.29	5.17	0.71	19.20	0.35	49.62	1433.54
100	Fillet of baked dory	100	101.77	3.66	0.00	0.00	17.16	0.00	74.80	500.01
101	Burgers with tomato sauce	101	186.80	11.83	1.23	1.23	18.77	0.36	51.51	1462.46
102	Stew with potatoes	102	272.70	11.50	21.98	2.72	21.40	2.50	72.15	1558.42
103	Meatballs with parsley	103	209.50	10.70	7.21	1.39	21.40	1.03	102.74	1500.36
104	Stew with vegetables	104	184.28	10.24	3.72	2.41	19.43	1.90	86.00	1454.81
105	Tomato and mozzarella salad	105	243.96	18.45	3.85	3.85	15.86	1.20	86.20	744.68
106	Fillet of baked dogfish	106	151.24	5.40	7.53	1.57	18.52	0.23	132.16	337.36
107	Turkey stew	107	162.00	9.72	1.65	1.65	17.07	0.52	70.15	216.05

108	Dogfish cutlet	108	145.91	4.51	6.88	1.78	19.80	0.27	176.44	361.74
109	Baked meatballs with tomato sauce	109	225.61	11.63	9.28	2.00	21.37	0.83	108.64	1510.25
110	Baked breaded cod sticks	110	240.45	7.10	26.85	0.60	18.75	2.40	78.50	578.35
111	Baked pork loin	111	143.95	7.88	0.00	0.00	18.18	0.00	68.40	399.85
112	Baked chicken and turkey sausage	112	170.95	10.70	0.00	0.00	18.60	0.00	71.00	196.35
113	Baked potato omelette	113	213.30	11.75	17.90	0.40	9.98	1.60	105.50	252.70
114	Butter and sage turkey breast	114	127.90	4.89	0.06	0.06	21.01	0.00	30.05	186.00
115	Slice of meat with tomato sauce	115	151.75	7.68	1.50	1.50	19.23	0.45	84.60	1449.85
116	Baked codfish balls	116	154.46	8.10	0.00	0.00	20.32	0.00	121.24	565.10
117	Tuna salad	117	150.10	5.62	11.28	2.51	14.20	1.68	195.50	246.56
118	Boiled meat with tomato sauce	118	175.15	10.38	1.50	1.50	18.87	0.45	52.20	1467.85
119	Meatloaf with milk sauce	119	243.56	17.10	0.53	0.53	21.80	0.00	99.19	1491.96
120	Baked anchovies with lemon	120	165.82	7.74	7.02	1.92	17.52	0.27	132.10	243.55
121	Tuna pie with potatoes and eggs	121	186.61	10.09	9.06	0.20	15.35	0.80	200.44	280.75
122	Omelette	122	131.55	11.14	0.23	0.23	7.60	0.00	84.25	205.30
123	Asiago cheese	123	179.50	12.80	0.40	0.40	15.70	0.00	380.00	334.85
124	Caciotta cheese	124	192.00	15.50	0.90	0.90	12.25	0.00	257.00	334.85
125	Crescenza cheese	125	196.70	16.31	1.33	1.33	11.27	0.00	245.00	235.20
126	Montasio cheese	126	205.50	16.10	1.00	1.00	15.15	0.00	378.50	334.85
127	Provolone cheese	127	187.00	14.10	1.00	1.00	14.05	0.00	430.00	334.85
128	Omelette	128	99.96	8.35	0.00	0.00	6.20	0.00	68.50	141.68
129	Baked beef burgers	129	175.30	9.48	3.50	0.23	19.10	0.17	69.81	1409.78
130	Baked pork burger	130	196.00	12.36	3.50	0.23	17.93	0.17	87.81	410.78
131	Breaded cod fillets	131	135.83	5.16	4.28	0.28	18.27	0.21	102.97	519.33
132	Breaded hake fillets	132	135.83	5.16	4.28	0.28	18.27	0.21	102.97	374.13
133	Breaded sea bass fillets	133	150.13	5.93	4.39	0.39	20.14	0.21	160.17	343.33
134	Buffalo mozzarella	134	187.20	15.86	0.26	0.26	10.86	0.00	130.00	631.80
135	Roasted chicken breast	135	125.96	4.72	0.00	0.00	20.97	0.00	29.70	175.88

136	Breaded chicken breast	136	172.91	5.95	7.00	0.45	23.37	0.34	82.14	217.28
137	Beef balls with tomato sauce	137	182.90	9.64	4.69	1.41	19.51	0.49	73.01	1451.83
138	Mixed meat balls with tomato sauce	138	206.96	11.97	4.99	1.71	20.05	0.58	100.91	983.74
139	Baked cod fillets meatballs	139	135.83	5.16	4.28	0.28	18.27	0.21	102.97	519.33
140	Baked beef meatloaf	140	175.30	9.48	3.50	0.23	19.10	0.17	69.81	1409.78
141	Baked ham	141	107.50	7.35	0.45	0.45	9.90	0.00	324.00	217.50
142	Baked ham with lasagna	142	53.75	3.68	0.23	0.23	4.95	0.00	162.00	108.75
143	Cured ham	143	134.00	9.20	0.00	0.00	12.75	0.00	1289.00	217.50
144	Cured ham with lasagna	144	67.00	4.60	0.00	0.00	6.38	0.00	644.50	108.75
145	Ricotta and cured ham	145	154.60	11.14	2.10	2.10	11.66	0.00	691.30	301.35
146	Slice of meat with cured ham	146	152.64	7.13	0.02	0.02	22.19	0.00	294.84	1444.06
147	Baked beef escalope	147	139.48	5.33	3.07	0.09	20.10	0.12	37.12	1406.40
148	Pork stew with rosemary	148	176.36	12.01	0.00	0.00	17.10	0.00	65.70	398.18
149	Strips of chicken with flour and broth sauce	149	139.60	4.76	3.05	0.07	21.43	0.12	29.78	181.72
150	Baked stips of beef	150	131.36	6.07	0.00	0.00	19.17	0.00	36.00	1397.18
151	Tuna with olive oil	151	96.00	5.05	0.00	0.00	12.60	0.00	158.00	200.00
152	Potatoe pie	152	342.60	17.17	31.96	2.44	16.86	2.57	252.04	596.28
153	Scrambled eggs	153	94.32	7.69	0.04	0.04	6.23	0.00	68.78	148.44
154	Chard with olive oil	154	70.45	5.15	4.20	4.20	1.95	1.80	15.00	38.35
155	Broccoli with olive oli	155	85.45	5.60	4.65	4.65	4.50	4.65	18.00	48.85
156	Roasted artichokes	156	77.95	5.30	3.75	2.85	4.05	8.25	199.50	425.35
157	Carrots with olive oil and lemon	157	80.25	5.20	7.67	7.67	1.11	3.10	95.10	23.85
158	Green beans with olive oil	158	71.95	5.15	3.60	3.60	3.15	4.35	3.00	83.35
159	Fennel gratin	159	76.72	5.62	3.83	1.65	2.77	3.41	30.00	44.54
160	Fried zucchini flowers with mozzarella and anchovy	160	200.00	9.04	23.28	0.32	5.92	0.96	2.16	222.40
161	Fennel salad	161	55.75	5.00	1.20	1.20	1.44	2.64	4.80	0.00
162	Tomato salad	162	65.35	5.24	3.36	3.36	1.44	1.20	3.60	65.95

163	Mixed salad with cucumbers (summer)	163	70.95	5.30	4.68	4.68	1.43	1.91	34.80	43.04
164	Mixed salad (winter)	164	71.15	5.18	4.84	4.84	1.55	2.55	42.70	28.36
165	Green salad	165	58.25	5.14	2.10	2.10	1.05	0.91	4.90	22.35
166	Mix of potatos carots and green beans	166	105.15	5.60	12.19	3.44	2.22	2.89	32.80	72.64
167	Baked potatoes	167	172.45	6.50	26.85	0.60	3.15	2.40	10.50	128.35
168	Potatoes with olive oil	168	172.45	6.50	26.85	0.60	3.15	2.40	10.50	128.35
169	Crispy baked potatoes	169	172.45	6.50	26.85	0.60	3.15	2.40	10.50	128.35
170	Roasted potatoes	170	172.45	6.50	26.85	0.60	3.15	2.40	10.50	128.35
171	Roasted peas	171	103.85	5.24	10.53	3.57	4.37	6.29	103.70	231.40
172	Mashed potatoes	172	231.97	10.92	29.05	2.80	6.04	2.40	56.21	235.88
173	Spinach with butter and Parmigiano	173	87.88	5.29	4.56	0.73	5.58	2.70	109.85	457.28
174	Spinach with olive oil	174	79.45	5.00	4.50	0.68	4.20	2.70	85.50	425.35
175	Zucchini gratin	175	75.49	5.23	5.21	2.15	2.35	1.95	49.00	72.35
176	Sautéed zucchini	176	61.45	5.15	2.10	1.95	1.95	1.80	33.00	68.35
177	Carrots with butter	177	90.40	4.47	11.46	11.46	1.69	4.65	142.85	38.25
178	Eggplant with tomato sauce	178	79.75	5.78	5.02	5.02	2.11	4.60	46.00	256.95
179	Peas with cured ham	179	135.32	6.37	11.56	3.73	8.72	7.02	503.08	328.89
180	Mixed vegetables gratin	180	70.38	3.81	6.47	4.23	2.98	2.96	55.21	170.69
181	Savoy cabbage with tomato	181	81.85	5.35	4.95	4.95	3.48	4.71	38.10	104.05
182	Baked asparagus with bechamel	182	117.54	5.48	9.23	6.26	8.47	3.50	40.26	136.88
183	Mushrooms with parsley	183	74.95	5.30	1.20	1.20	5.55	1.05	7.50	158.35
184	Green beans with tomato sauce	184	80.35	5.35	4.80	4.80	3.63	4.71	6.60	138.55
185	Peppers gratin	185	57.16	2.96	6.44	6.44	1.59	3.29	53.91	176.50
186	Peppers with olive oil	186	77.95	5.45	6.30	6.30	1.35	2.85	3.00	173.35
187	Roasted new potatoes	187	149.95	5.45	23.55	0.30	3.00	2.10	15.00	128.35
188	Baked ootatoes croquette	188	205.89	7.65	30.81	0.92	5.19	2.59	47.36	177.56
189	Chickpeas with tomato sauce	189	242.95	8.98	29.34	3.42	13.02	8.52	7.20	93.55
190	Cauliflower with anchovy sauce	190	89.30	5.45	4.17	3.72	6.05	3.60	192.20	30.20

191	Baked cauliflower with bechamel	191	109.54	5.52	8.08	4.66	7.29	3.72	45.76	82.38
192	Fennel with butter	192	66.88	5.29	1.56	1.56	3.18	3.30	30.35	61.73
193	Bread	193	110.00	0.20	25.40	0.80	3.24	1.52	117.20	40.00
194	Fruits	194	63.33	0.20	15.48	15.48	0.89	2.70	2.67	47.40
